# Outcomes of surgical treatment of patellar instability in children with Down syndrome

**DOI:** 10.1186/s13018-024-04730-y

**Published:** 2024-04-25

**Authors:** Assem Zein, Alaa Zenhom Mahmoud Hassan, Amr Mohamed Soliman, Mohamed Mohamed Azmy Mohamed

**Affiliations:** 1 Department of Orthopaedics and Trauma Surgery, Minia University, Minia, Egypt; 2 Minia University, Minia, Egypt

**Keywords:** QT autograft, MPFL reconstruction, Patellar instability, Down syndrome

## Abstract

**Background:**

patellar instability is a relatively frequent musculoskeletal disorder in children with Down syndrome (DS). However, such a condition has seldom been studied in the literature, even less its surgical treatment. Different techniques have been offered for this condition; the evidence for surgical options is scarce and primarily based on case reports or case series with few patients and heterogeneous techniques. Given this background, we aimed to evaluate the outcomes of a uniform kind of surgical procedure for such a condition that combined lateral soft tissue release, medial patellofemoral ligament (MPFL) reconstruction (using a partial-thickness quadriceps tendon autograft), the Roux-Goldthwait procedure, and V-Y quadricepsplasty (if needed).

**Materials and methods:**

This retrospective study involved 11 skeletally immature patients (12 knees; 9 males and 2 females), 5.5 to 14.1 years of age, with DS who had patellofemoral instability (PFI) and were managed by this technique between October 2018 and March 2020. Preoperative radiography, CT scan, and MRI were performed to evaluate the physis status, lower limb alignment, patellar height, trochlear morphology, and any associated knee pathology. A functional knee assessment was done by using the Kujala score and the modified Lysholm score.

**Results:**

The mean time of follow-up (± SD) was 47.7 ± 5.8 months (range: 39–56). Pre-operatively, the Kujala score (± SD) was 52.6 ± 14.3 (range: (31–74), and at final follow-up, it was 92.2 ± 4.4 (range: (88–98), showing a significant improvement (*P* < 0.001). The preoperative modified Lysholm score (± SD) was 54.3 ± 8.1 (range: 39–62), and at final follow-up it was 92.4 ± 5.3 (range: 82–96), showing a significant improvement (*P* < 0.001). All patients had a stable patella without a recurrence of instability and regained full ROM. There was no incidence of a patellar fracture or femoral physis injury.

**Conclusions:**

Our proposed technique of combined soft tissue procedures, including lateral soft tissue release, MPFL reconstruction (using a partial-thickness quadriceps tendon autograft), the Roux-Goldthwait procedure, and V-Y quadricepsplasty, was an effective method for treating patellar instability in children with DS while avoiding physeal injury and patellar fracture. Functional scores and radiological outcomes were improved.

**Level of evidence:**

IV; retrospective case series.

## Introduction

Down syndrome (DS) is the most common chromosomal abnormality in humans and typically results from a maternal duplication of chromosome 21, resulting in trisomy 21 [1]. Its prevalence in the United States is estimated at 1 in 700–1,000 live births [2]. The increased ligamentous laxity and muscle hypotonia (associated with DS) may be linked to an increased quantity of type VI collagen, which is partially coded by genes on chromosome 21 [3]. A variety of musculoskeletal disorders are encountered in children with DS and are thought to be related to generalized ligamentous laxity, muscle hypotonia, and joint hypermobility, presenting a great challenge for treatment [4].

The prevalence of patellofemoral instability (PFI) is 10–20% in these patients [4]. The life expectancy of patients with DS has improved to about 50 years [5]. As a result, there seems to be an increasing need for early diagnosis and appropriate treatment of instances exhibiting PFI.

Even though PFI is relatively common in DS patients, there hasn’t been much written on the disorder or its surgical management in the literature. Different procedures have been proposed for this condition; however, the evidence for surgical alternatives is sparse and mostly based on case reports or case series with few patients and heterogenous techniques [6–8]. . The clinical picture, postoperative compliance, and expectations are different from those in children without Down’s syndrome. A multidisciplinary and multisystem approach should accompany any surgical treatment. Combined techniques are needed to address this unique condition [9–13].

The medial patellofemoral ligament (MPFL) is the main lateral restrictor of the patella Although many studies showed good results with MPFL reconstruction techniques in children and mainly adults [14], very few studies were conducted on MPFL reconstruction in children with DS. There is a great concern when performing MPFL reconstruction in skeletally immature patients because of the possibility of femoral physis damage and/or fracture of a proportionately smaller patella. The concern becomes even greater in children with DS due to the associated patellar hypoplasia, trochlear dysplasia, ligamentous hyperlaxity, muscle hypotonia, joint hypermobility, intellectual deficit, and associated systemic diseases.

**Fig. 1:**
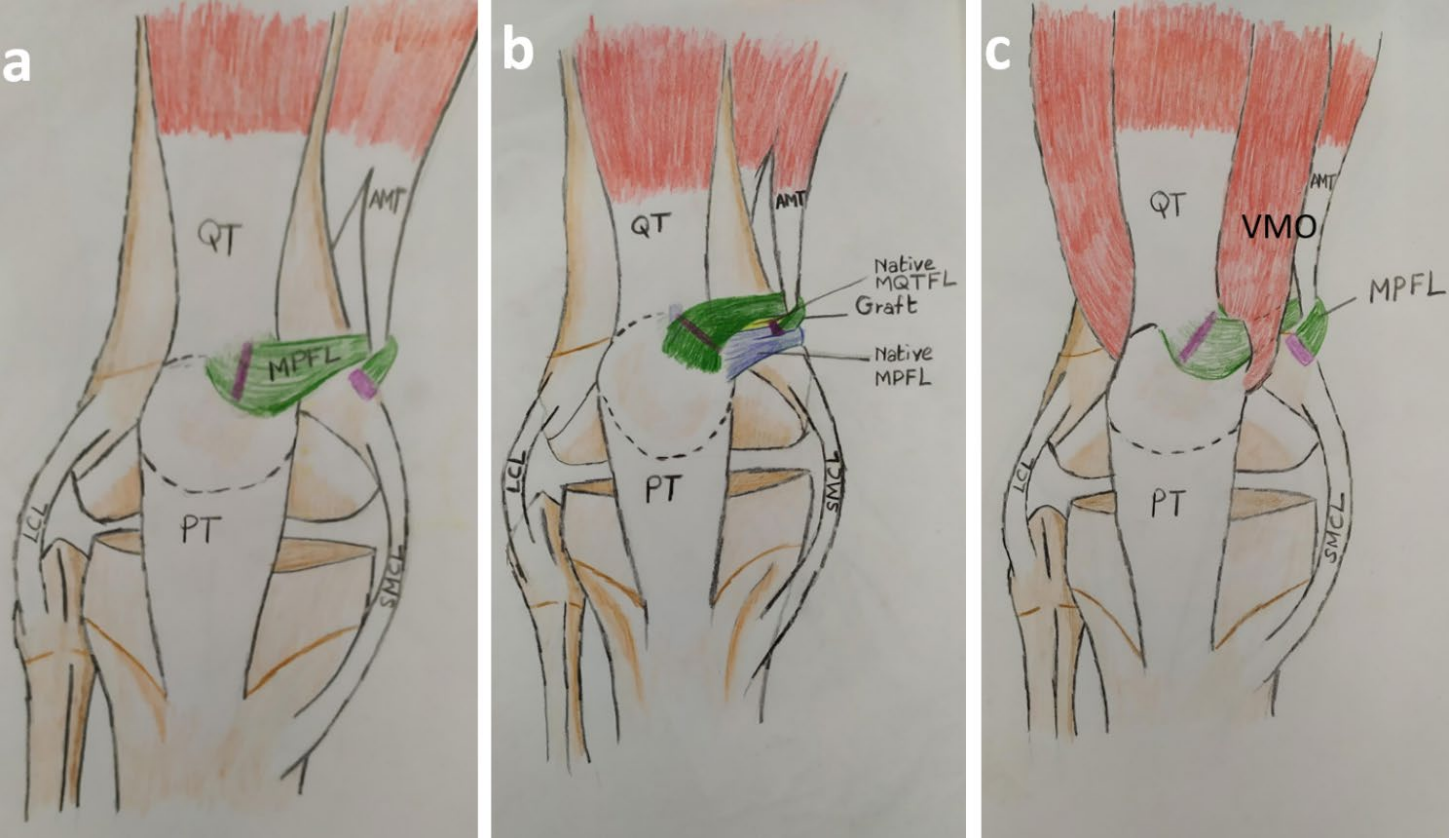
Post-operative schematic diagram of the knee joint. (**A**) Diagram in the absence of the VMO: The MPFL graft is depicted as being reflected medially and looped around the AMT near its insertion. (**B**) Diagram depicting the MPFL graft (green color) and its relationship to the native MQTFL (yellow color) and the native MPFL (blue color). (**C**) Diagram in the presence of VMO showing the MPFL graft as being passed deep to the VMO; the pink lines represent the sites of sutures (at the anterior surface of the patella and at the femoral origin, which is distal and anterior to the AT). QT, Quadriceps Tendon; MQTFL, Medial Quadriceps Tendon Femoral Ligament; MPFL, Medial Patellofemoral Ligament; PT, Patellar Tendon; VMO, Vastus Medialis Obliquus; AMT, Adductor Magnus Tendon; LCL, Lateral Collateral Ligament; SMCL, Superficial Medial Collateral Ligament

Nearly most of the previously described MPFL reconstruction techniques either use patellar bony sockets, tunnels (single or double), or longitudinal slots with anchors that could place the proportionately smaller and hypoplastic patellae in children with DS at higher risk of fracture [15]. Similarly, most techniques use femoral tunnels or sockets for intraosseous femoral graft fixation that place the femoral physis and articular cartilage at risk of injury [14]. Although the soft tissue pulley technique does not place open femoral growth plates at risk, it does not allow for anatomic MPFL reconstruction and has inferior clinical results [16].

we aimed to evaluate the outcomes of a uniform kind of surgical procedure for PFI in children with associated DS. The conducted procedure combined lateral soft tissue release, MPFL reconstruction (Fig. 1), the Roux-Goldthwait procedure, and V-Y quadricepsplasty (if needed).

We hypothesized that surgical treatment of PFI in children with DS would improve the functional scores of this category of patients. A combination of soft tissue techniques, including MPFL reconstruction, would be needed to centralize the patella and restore its stability. A hardware- and drill-free anatomic MPFL reconstruction using a partial-thickness QT autograft would help to restore patellar stability while avoiding patella fracture and femoral physis injury.

## Patients and methods

### Patients

Fifteen skeletally immature patients with Down syndrome who had PFI and were managed by this combined procedure from October 2018 to March 2020 were reviewed for a retrospective study (Table 1). Human ethical committee approval with number (No. 960 − 11/2023) and patients’ full informed consents were obtained.

Inclusion criteria:

- Only open-physis patients with Down syndrome who had symptomatic patellar instability and were unimproved despite a non-operative treatment program were eligible for inclusion in the study.
- Minimum follow-up of 2 years.

Exclusion criteria:

- Patellar instability in the absence of Down syndrome.
- Skeletally mature patients.
- Patients with severe abnormalities in the coronal plane or malrotation of the leg.

Patella alta (Caton-Deschamps > 1.2) and increased tibial tubercle trochlear groove distance (TT-TG > 20 mm) were no contraindications.

After excluding cases of lost follow-up (two cases) and cases with incomplete records (two cases), 11 patients (9 males and 2 females) were eligible for the study. Surgery was performed bilaterally in one case. The mean age at surgery (± SD) was 10.7 ± 2.4 years (range: 5.5–14.1). Six right and six left knees were operated on. No patient had had previous knee surgery. The diagnosis of DS was made clinically by the presence of the classic clinical features of DS, such as oblique palpebral fissures, epicanthal folds, and a flat facial profile. Confirmation of the diagnosis was made by a formal karyotype [17].

According to the Dugdale classification of patellar instability [6], 5 patients had grade III patellar instability, 4 patients (5 knees) had grade IV, and 2 patients had grade V patellofemoral instability. The most common symptoms at initial presentation were frequent falls during daily activities (in all 11 patients), pain (4 patients; 5 knees), and limping (7 patients). According to the Dejour classification of trochlear dysplasia [18], the trochlea was Dejour type A in 3 (25%) knees, Dejour type B in 4 (33.3%) knees, Dejour type C in 3 (25% knees), and Dejour type D in 2 (16.7%) knees (Table 1).

**Table 1:** Information and parameters about the cohort

CASE	Age at surgery In (years + months)	sex	Side	Prior knee surgery type	Final follow up in months	Main Symptom	Dugdale Grade	Caton Dechamp Ratio	TT-TG distance in mm	MAD		PTA		MPFL Length in mm	Trochlear Dysplasia Dejour	Surgical procedures	Kujala score		Modified Lysholm	
										Pre	Final	Pre	Final				Pre	Final	Pre	Final
1	5 + 6	M	R	No	56	Frequent Fall Limping	3	1.10	12	+ 1.13	+ 1.7	25.6	2.4	79	B	LR+ (R-G) procedure + MPFLR	74	88	62	91
2	12	M	R	No	54	Frequent Fall Limping	4	1.50	14	+ 1.21	+ 1.11	35.6	3.5	89	B	LR+ (R-G) procedure + MPFLR	55	96	53	96
3	14 + 1	M	R	No	53	Frequent Fall Pain	3	1.10	17	+ 1.64	+ 1.84	24.1	2.6	85	A	LR+ (R-G) procedure + MPFLR	61	96	62	96
4	8	F	L	No	52	Frequent Fall Limping Pain	5	1.10	22	+ 3.31	+ 2.23	44.6	1.8	83	C	LR + VY Q lengthening + (R-G) procedure + MPFLR	55	88	54	82
5	11	M	L	No	52	Frequent Fall Pain	4	1.00	21	+ 1.31	+ 1.25	27.2	3.9	82	D	LR+ (R-G) procedure + MPFLR	61	96	62	96
5	11 + 8	M	R	No	44	Frequent Fall Pain	4	1.10	13	+ 1.33	+ 1.23	26.9	2.1	83	C	LR+ (R-G) procedure + MPFLR	63	98	62	96
6	13	M	L	No	49	Frequent Fall Limping	3	1.10	14	+ 0.3.7	+ 3.21	23.6	3.3	82	B	LR+ (R-G) procedure + MPFLR Loose body removal+	31	88	49	82
7	10 + 5	F	R	No	47	Frequent Fall Limping	4	0.95	11	+ 3.66	+ 3.44	26.3	2.7	80	C	LR+ (R-G) procedure + MPFLR	55	96	54	96
8	9 + 3	M	L	No	45	Frequent Fall	3	1.00	12	+ 4.45	+ 5.41	24.2	2.8	78	A	LR+ (R-G) procedure + MPFLR	51	96	39	96
9	9 + 2	M	R	No	42	Frequent Fall Limping	5	0.90	15	+ 4.33	+ 3.11	39.4	2	90	B	LR+ (R-G) procedure + MPFLR	63	88	49	91
10	11 + 1	M	L	No	40	Frequent Fall Limping	4	1.00	20	+ 1.39	+ 1.01	27.1	1.3	92	D	LR+ (R-G) procedure + MPFLR + MM repair.	31	88	43	91
11	13 + 2	M	L	No	39	Frequent Fall Pain	3	1.00	8	+ 5.23	+ 6.1	22.6	1.7	92	A	LR ++ (R-G) procedure + MPFLR + Patellar chondroplasty + LM repair	31	88	62	96
Range ±SD	(5.5–14.1) 10.7 ± 2.4				(39–56). 47.7 ± 5.8			(0.9–1.5) 1.1 ± 0.2	(8–22 mm). 14.9 ± 4.3 mm	Median 2.48 IQR (1.32–4.17)	Median 2.04 IQR (1.24–3.38)	(22.6–44.6) 28.9 ± 7	(1.3–3.9) 2.5 ± 0.8	(78–92) 84.6 ± 5			(31–74) 52.6 ± 14.3	(88–98) 92.2 ± 4.4	(39–62). 54.3 ± 8.1	(82–96) 92.4 ± 5.3

M, Male; F, Female; R, right; L, left.; TT-TG distance, Tibial Tubercle -Trochlear Grove distance; MAD, mechanical axis deviation; PTA, patellar tilt angle; R-G, Roux Goldthwait procedure; LR, Lateral soft tissue Release; MPFLR, Medial patellofemoral ligament reconstruction; Q, Quadriceps; LM, lateral meniscus; MM, medial meniscus.

### Methods

#### Surgical technique

**Fig. 2:**
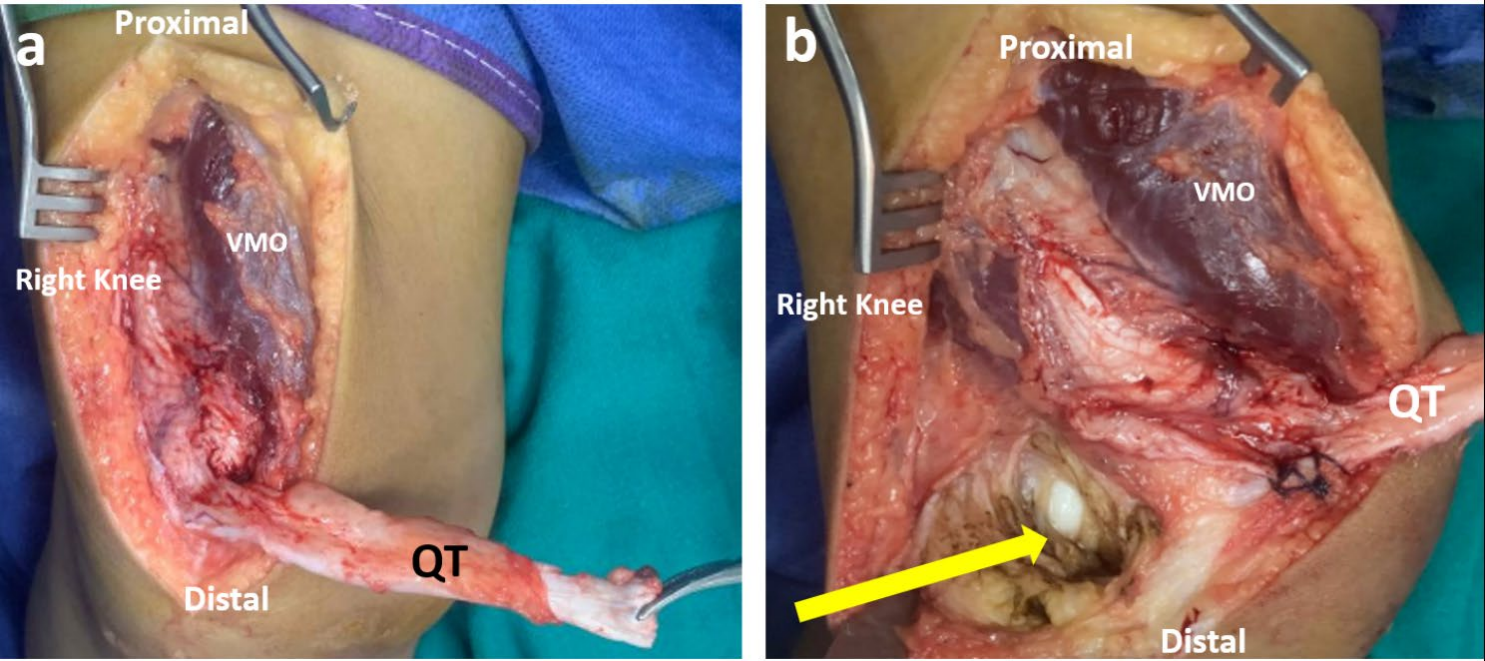
Intraoperative photo of the right knee. (**A**) A partial–thickness quadriceps tendon graft is harvested, measuring about 8–9 cm long, 10–15 mm wide, and about 2–3 mm thick. The graft is released proximally while remaining intact distally at the anterior surface of the patella. (**B**) Release of the contracted lateral soft tissues (yellow arrow) to centralize the patella

All surgeries were performed by a single senior surgeon (Z.A.M.). For this study, we adopted a modification of the treatment technique described by Zein et al. for MPFL reconstruction [19]. . In brief, an arthroscopic knee examination was performed and included management of any intra-articular chondral or meniscal pathology as well as evaluation of patellar tracking. An anterior midline skin incision was first made, originating about 6–8 cm proximal to the patella and extending to about 2–3 cm below the tibial tuberosity. A partial-thickness quadriceps tendon graft was then harvested, approximately 10 to 15 mm wide, 8 to 9 cm long, and 2 to 3 mm thick. The graft was released from proximal to distal until the anterior surface of the upper third of the patella (Fig. 2A). It was secured to the soft tissue on the anterior surface of the patella by a non-absorbable stitch. The length of the QT graft was assessed by reflecting it across the medial side of the knee to see if it could reach the medial epicondyle. The tendon could be lengthened if it was short by longitudinal splitting of the tendon, releasing the distal attachment of one half of the tendon while leaving its proximal attachment intact on the other half, and then reflecting the released half to 180^0^. An extensive lateral release of the contracted soft tissues and synovium was performed to centralize the patella in the trochlear groove (Fig. 2B).

**Fig. 3:**
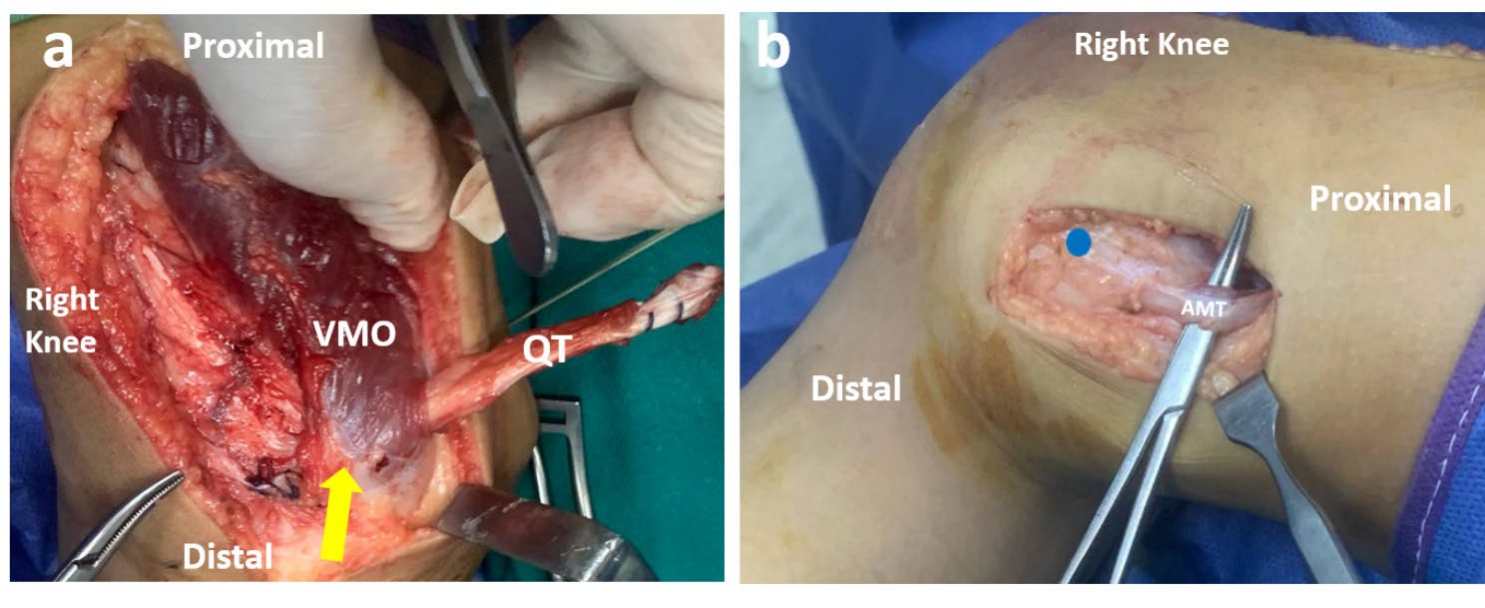
An intraoperative photo of the right knee. (**A**) Passage of the graft deep to the VMO (yellow arrow). (**B**) identification of AMT at its femoral insertion. The blue dot represents the anatomic femoral attachment of the native MPFL, which is distal and anterior to the adductor tubercle. AMT, adductor magnus tendon; QT, quadriceps tendon; VMO, vastus medialis obliquus; MPFL, medial patellofemoral ligament

Then the QT graft was passed medially deep to the vastus medialis obliquus muscle (VMO) with care to remain extra-articular (Fig. 3A). The attention was then directed to the medial side of the knee. A 2–3 cm skin incision was centered on the AT (Adductor Tubercle). The adductor magnus tendon (AMT) was identified at its insertion near the AT. The insertion of the AMT on the AT was used as an intra-operative anatomic landmark for the precise localization of the femoral attachment of the MPFL, which is just distal and anterior to the AT [20]. (Fig. 3B).

**Fig. 4:**
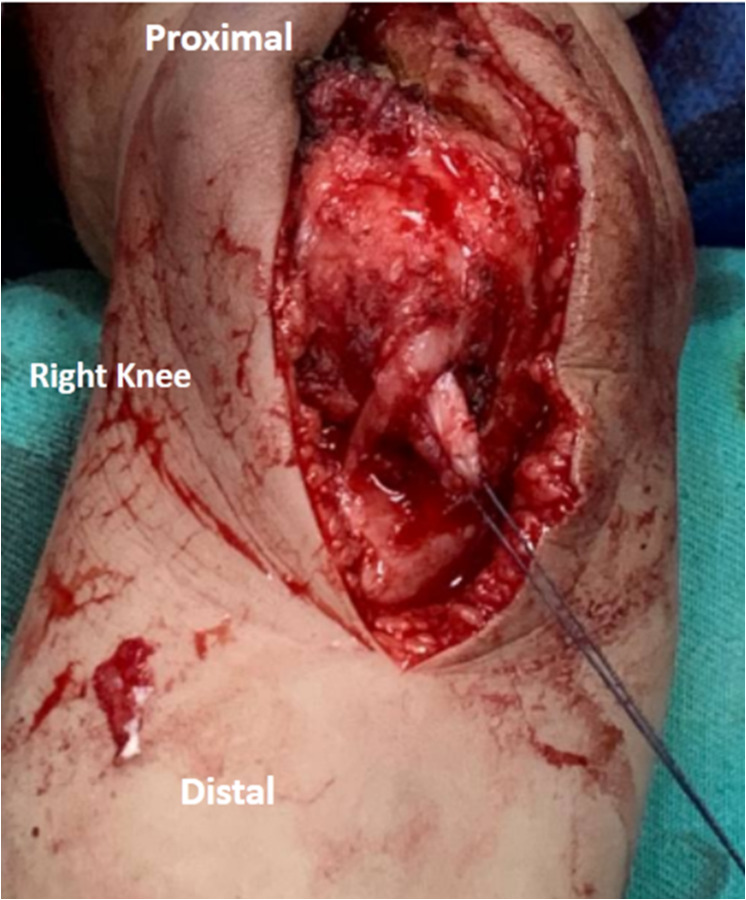
An intraoperative photo of the right knee showing the Roux-Goldthwait procedure. The patellar tendon was split longitudinally into two halves. The lateral half was released at its distal bony attachment, and it was then placed under the medial half and reattached to the soft tissue on the medial side of the proximal tibia

The Roux-Goldthwait procedure was used to distally re-align the extensor mechanism. The patellar tendon was split longitudinally into two halves. The lateral half was released at its distal bony attachment, and it was then placed under the medial half and reattached to the soft tissue on the medial side of the proximal tibia (Fig. 4).

**Fig. 5:**
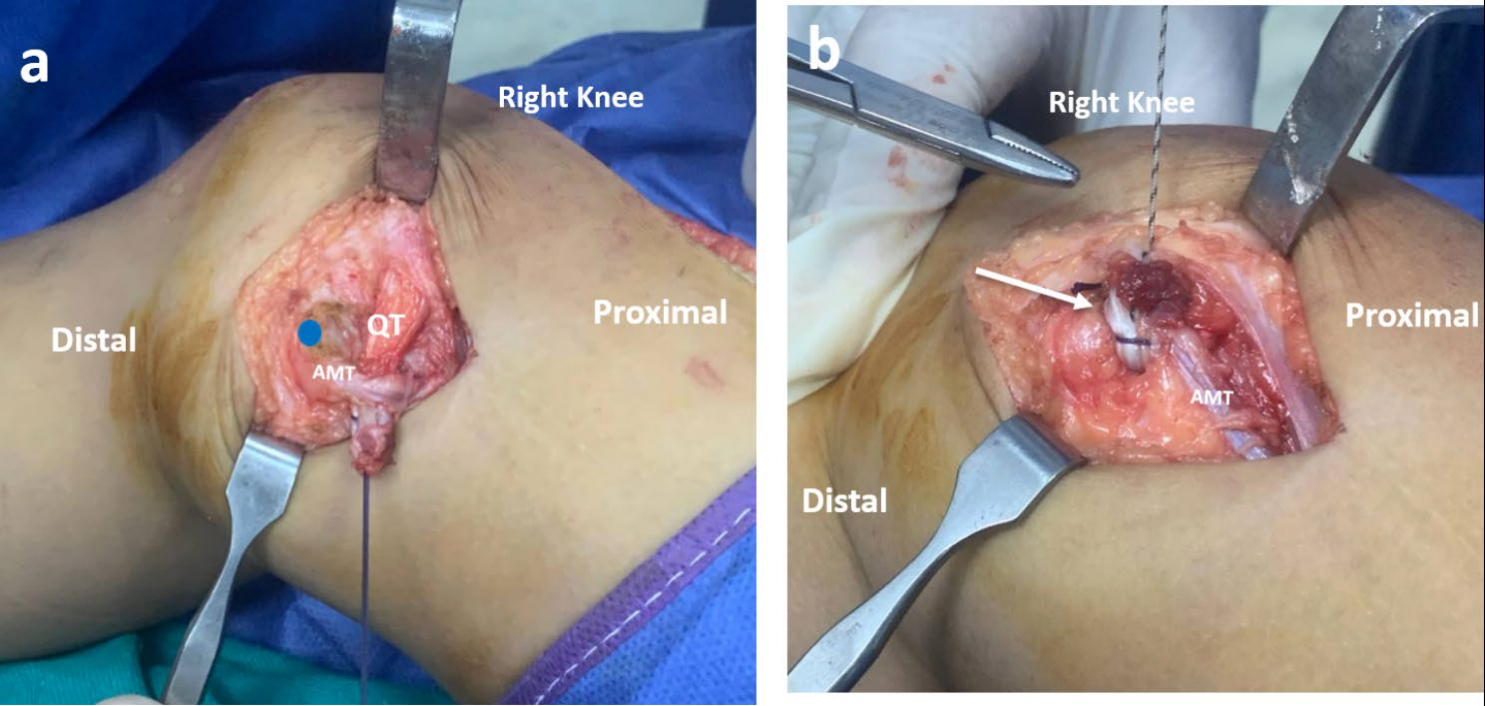
An intraoperative photo of the right knee. (**A**) Passage of the QT graft deep to the AMT near its insertion. The blue dot represents the anatomic femoral attachment of the native MPFL, which is distal and anterior to the adductor tubercle. (**B**) Fixation of the QT graft with non-absorbable sutures to the anatomic femoral origin of the native MPFL with the knee in 40^0^-60^0^ flexion and the patella centralized in the trochlear groove

At the AMT insertion, the graft was passed deep to the AMT. Then the graft was turned around the AMT insertion, which acts as a pulley. The free end of the MPFL graft was finally sutured to the thick periosteum and soft tissue on the native MPFL’s femoral attachment, which is distal and anterior to the AT with the knee in 40^0^-60^0^ flexion (Fig. 5A and B).

### Postoperative care

The limb was immobilized in an above-the-knee long cast with a 20^0^ to 30^0^ of knee flexion for 4 weeks without weight bearing. Range of motion and strengthening exercises were started after cast removal. Then, gradual, protected weight bearing commenced. As soon as the patient regained muscular control of the quadriceps, non-protected weight-bearing was permitted.

### Methods of assessment

**Fig. 6:**
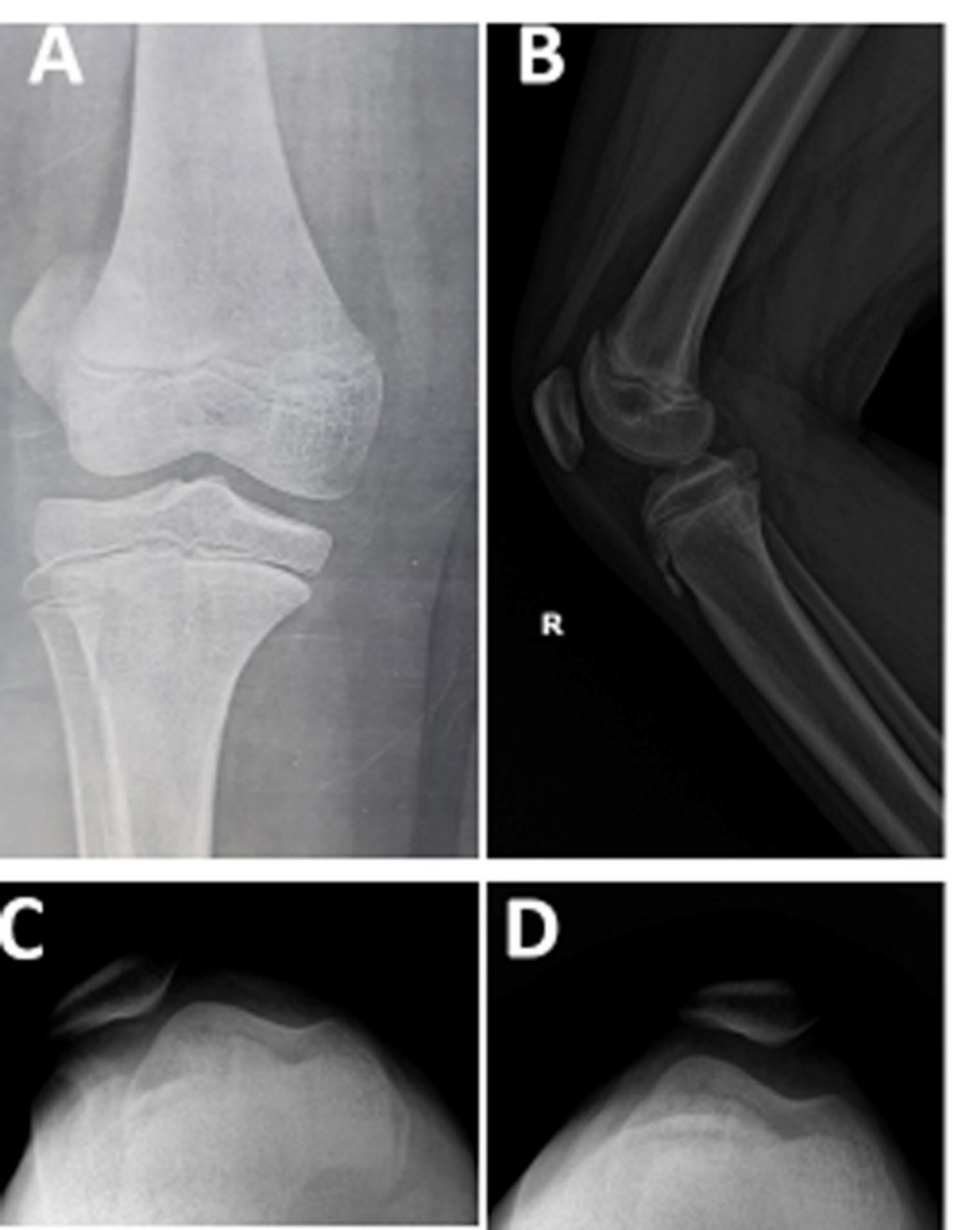
Preoperative and postoperative radiography. (**a**) True antero-posterior view. (**b**) True lateral view of the right knee. (**c**) Axial view of the right knee in 40^0^ knee flexion, showing a laterally dislocated patella. (**d**) Postoperative axial view of the same knee in 40^0^ knee flexion, showing a well-reduced patella

**Fig. 7:**
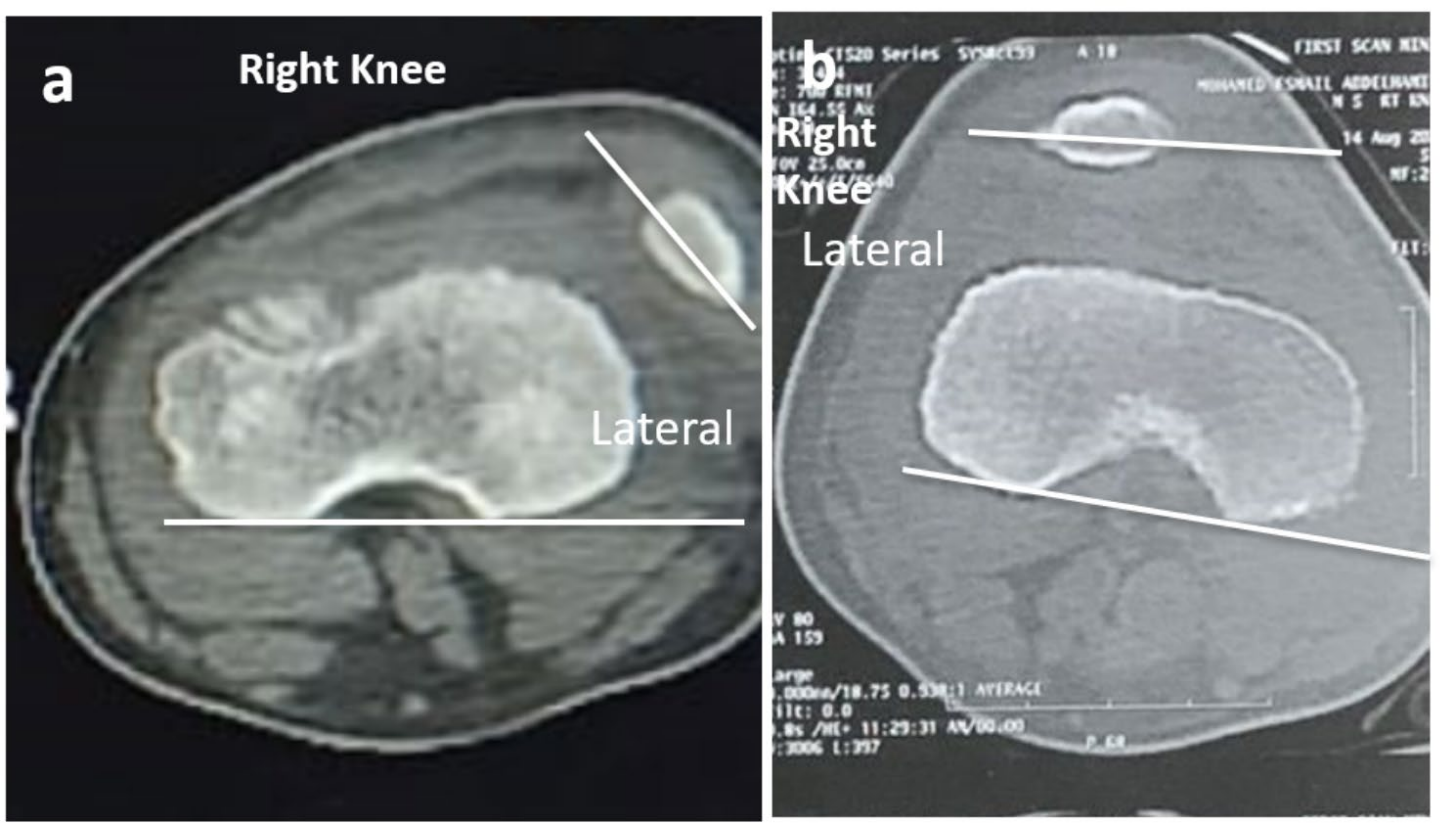
Computed Tomography CT scans. (**a**) Preoperative axial CT scan of the right knee showing lateral patellar subluxation and the patellar tilt angle (PTA; the angle formed between a line defining the maximal width of the patella and the femoral posterior condylar line; 44.6^0^). (**b**) Post-operative final axial CT scan of the same knee showing an improved PTA (1.8^0^) and a centralized patella

Preoperative and postoperative knee assessment included assessment of a patient’s symptoms and clinical evaluation of the patient’s gait, patella location, presence of the J sign, patella apprehension, and range of motion. The lateral patella glide test was employed to assess lateral patella displacement before and after MPFL reconstruction, in which the patella is divided into four vertical quadrants. A lateral patella glide that is normal should not exceed 2 quadrants [21]. Further, radiological evaluation included pre-operative and post-operative radiographs (standard AP view, true lateral view, axial view, and long film standing view), MRI, and 3D-CT (Figs. 6 and 7). The functional outcomes were assessed preoperatively and at final follow-up using the Kujala score [22] and the modified Lysholm score [8].

### Statistical method

The analysis of the data was carried out using the IBM SPSS version 25 statistical package software (Armonk, NY, USA). The normality of the distribution of the data was determined by the Shapiro-Wilk test. Data were expressed as mean ± SD and minimum and maximum of range for parametric quantitative data and by both number and percentage for qualitative data. Analyses were done between the two times (preoperatively and at the final follow-up) for parametric quantitative data using the Paired Sample T test. A P-value < 0.05 was considered statistically significant.

## Results

The mean period of follow-up (± SD) was 47.7 ± 5.8 months (range: 39–56). Details of the cases are summarized in Table 1. Patellar height by the Caton-Dechamp ratio was 1.1 ± 0.2 (0.9–1.5). The mean TT-TG (± SD) was 14.9 ± 4.3 mm (range: 8–22 mm). Two cases had a TT-TG greater than 20 mm. The pre-operative MAD median was 2.48 (IQR, (1.32–4.17)) and at final follow-up, it was 2.04 (IQR, (1.24–3.38)), showing a non-significant change (*P* = 0.530). The pre-operative patellar tilt angle (PTA) (± SD) was 28.9 ± 7 degrees (range: 22.6–44.6) and at final follow-up was (± SD) 2.5 ± 0.8 (range:1.3–3.9), showing a significant improvement (*P* < 0.001). The MPFL length (± SD) was 84.6 ± 5 mm (range (78–92 mm).

Among the four patients (five knees) with pre-operative pain, complete relief was achieved in three patients, while one patient had mild pain with severe exertion that was relieved by rest and infrequent analgesics. The episodes of falls during daily activities disappeared or decreased in all the cases (sporadic episodes of falls continued in two cases during running). None of the patients had limping at the final follow-up. No recurrence of dislocation was recorded in any case, and all the cases had a normal passive lateral patellar glide at the final follow-up. However, the sporadic episodes of falls that were recorded in two cases could be attributed to episodes of subluxation with spontaneous reduction.

Concomitant procedures are shown in Table 2. Loose body removal was done in one case, meniscal repair was done in two cases, V-Y quadriceps lengthening was done in one case, and patellar chondroplasty was done in one case.

At the final follow-up, all the knees regained full ROM. Ten patients regained full quadriceps muscle power and size, while one patient had quadriceps muscle wasting (10 mm in comparison to the sound side).

**Table 2:** Type and distribution of concomitant procedures

Concomitant procedures		Descriptive statistic *N* = 12
**Loose body removal**	No Yes	11(91.7%) 1 (8.3%)
**Meniscus repair**	No Yes	10 (83.3%) 2 (16.7%)
**Patellar chondroplasty**	No Yes	11(91.7%) 1(8.3%)

N = Number

**Fig. 8:**
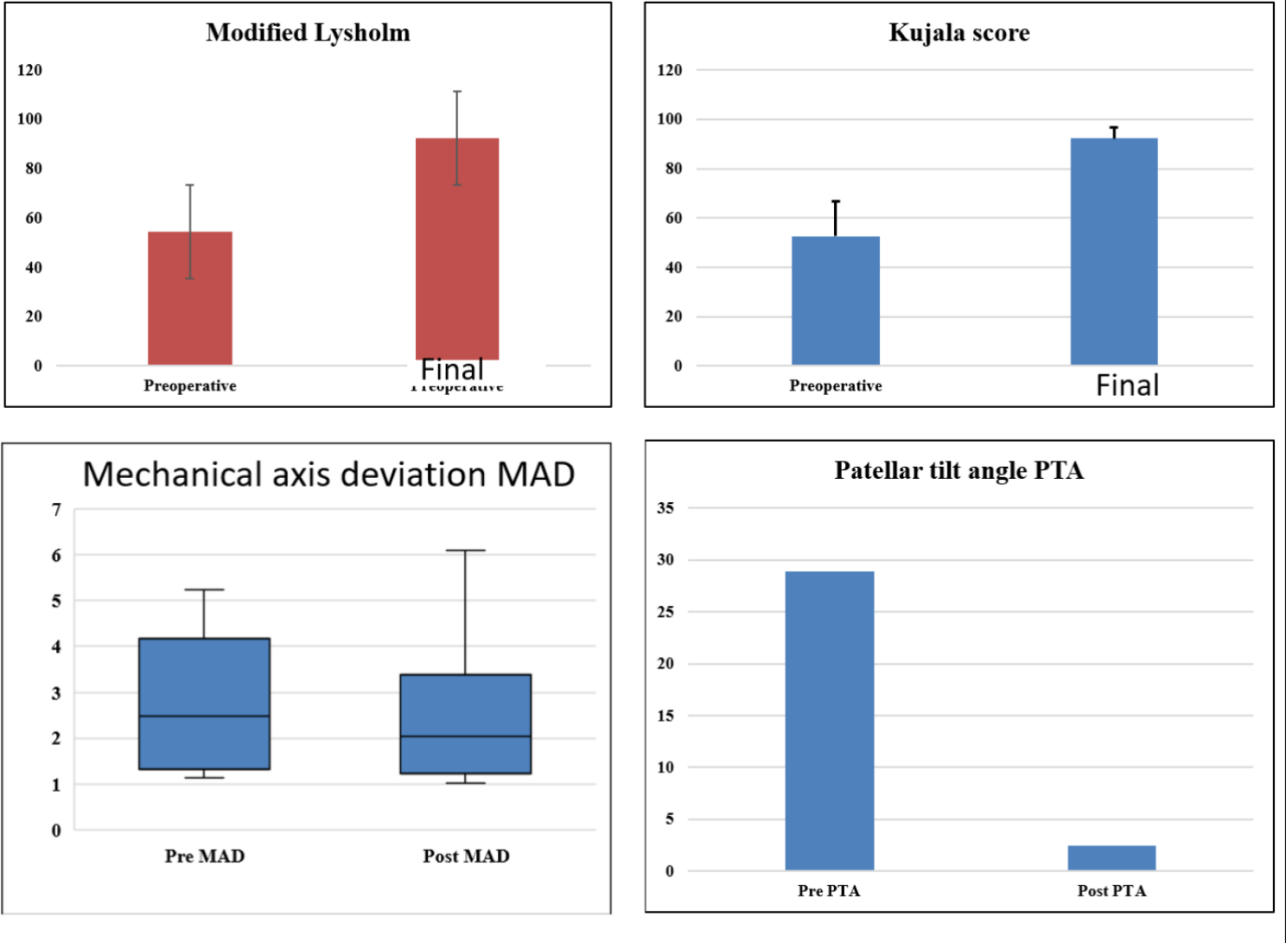
Preoperative and final follow-up functional scores and radiographic parameters. Preoperative and final follow-up modified Lysholm score (top left). Preoperative and final follow-up Kujala score (top right). The mechanical axis deviation MAD (bottom left) and the patellar tilt angle PTA (bottom right)

The preoperative Kujala score (± SD) was 52.6 ± 14.3 (range: (31–74) and at final follow up (± SD) was 92.2 ± 4.4 (range: (88–98) showing a significant improvement (*P* < 0.001) (Table 3; Fig. 8). The preoperative modified Lysholm score (± SD) was 54.3 ± 8.1 (range: 39–62).and at final follow up (± SD) was 92.4 ± 5.3 (range: 82–96) showing a significant improvement (*P* < 0.001) (table 3) (Fig. 8).

**Table 3:** Comparison of pre-operative and at final follow-up Kujala knee function score and modified Lysholm score

		Preoperative	At final follow-up	P value
		*N* = 12	*N* = 12	
**Kujala score**	*Range* *Mean ± SD*	(31–74) 52.6 ± 14.3	(88–98) 92.2 ± 4.4	< 0.001*
**Modified Lysholm**	*Range* *Mean ± SD*	(39–62) 54.3 ± 8.1	(82–96) 92.4 ± 5.3	< 0.001*

N = Number; P value, significance level of *p* < 0.05; SD, standard deviation

### Complications

Few complications were observed in the present series. One patient had knee effusion that improved with medical treatment. A hypertrophic wound scar was encountered in two patients; however, no further intervention was necessary. One patient had quadriceps wasting that required an intensive and prolonged course of rehabilitation. sporadic episodes of falls continued in two cases during running. One patient had mild pain with exercises that was relieved by rest and infrequent analgesics. No patellar fracture, recurrence of instability, or femoral physeal injury were encountered.

## Discussion

The results of this study showed the effectiveness and safety of the proposed technique, with a significant improvement in functional scores. Patellar stability was restored in all patients. No patellar fracture, physeal injury, or recurrence of patellar dislocation were seen in this series.

Patellar instability associated with DS has a specific nature, as it is related to ligamentous laxity, muscle hypotonia, and joint hypermobility. In addition, this population of patients has associated intellectual and systemic disorders. This presents a considerable challenge for management [7]. Children and parents must be advised of the abnormal nature of their collagen and that surgical interference may not overcome this genetic predisposition to instability.

The clinical presentation of patellofemoral instability in patients with DS might range from no symptoms at all to frequent falls, limbing, and pain. Indications for surgical stabilization of patellar instability in patients with DS have been questioned due to the frequent absence of symptoms or functional problems. Some authors considered this type of instability as not necessarily disabling and that there is no direct correlation between the degree of instability and functional disability [6, 7]. However, our study, in accordance with other studies, showed that the more severe grades were associated with a worse functional score (limitation of activities due to a weak extensor mechanism with frequent falls) [ 23]. Furthermore, all the patients in our study had significant symptoms in the form of frequent falls, pain, and limbing. Additionally, long-standing fixed lateral patellar dislocation and habitual dislocation result in knee deformation and arthritis if left untreated [8, 23, 24]. Moreira et al. [25] found in their study regarding trochlear dysplasia and patellar instability in patients with DS that trochlear normal development is dependent on PF stability. They concluded that patients with PF instability had trochlear dysplasia, while those with PF stability had normal trochlea.

We have detected pain in four (41.6%) of the eleven patients with dislocatable, dislocated reducible, and dislocated irreducible patellae; pain was reported by Menez et al. [9]. in 44% and by Dugdale et al. [6]. in 25% of knees with irreducible dislocated patella. Pain is occasionally taken into scant account in patients with DS, as these individuals do not usually display signs of distress equal to the general population. Previous studies [26] have shown that these patients are sensitive to pain but express it more slowly and inaccurately than the normal population. Consequently, we have to take these findings into consideration for both pre-operative evaluation of this category of patients and also for post-operative pain control procedures, even in the absence of obvious pain manifestations.

Caird et al. [27] recommended that conservative treatment in the form of physiotherapy or bracing can be tried in the initial phases of instability (grade II) that have no pain and scarce functional disability. They also recommended surgical treatment for symptomatic immature patients that included lateral soft tissue release and medial reefing.

Due to the complex nature of the condition, surgical management of patellar instability in patients with DS is complex, and a variety of surgical techniques is required to address it. Evidence for surgical options is limited and is primarily based on case reports or case series with few patients and differing techniques [6–8].

In terms of the number of surgical cases and procedures employed, Dugdale and Renshaw [6] operated on five patients (eight knees) with differing techniques for each patient, with only 50% of good results. A retrospective study by Mendez et al. [7] presented 14 surgeries in ten patients through lateral release and imbrication of the medial capsule. In more severe cases, the release was advanced proximally until the insertion of the vastus lateralis. The vastus medialis was advanced distally and laterally over the patella in 11 knees. A medial transfer of the patellar tendon insertion was performed additionally in 11 knees. The procedure was effective in restoring function and stability; however, it did little to correct deformities and prevent degenerative changes in grades 4 and 5. In 2007, Joo et al. [28] presented the results of a four-in-one procedure in five patients (six knees) with severe generalized laxity. Down syndrome was present in only two cases. It included, lateral soft tissue release, Z-plasty lengthening of the vasus lateralis, semitendinosus tenodesis to the patella using a non-absorbable anchor screw, proximal tube realignment of the patella, and medial transfer of the lateral half of the patellar tendon. Bettuzzi et al. [8], in 2009, performed the Roux-Goldthwait- Campbell technique on six children (10 knees). In 2012, Kocon [29] performed quadricepsplasty in eight patients (10 knees) using the technique modified by Green associated with semitendinosus tenodesis in older patients (modified Galeazzi), with two cases of failure. Finally, the modified Langenskiold procedure was used by Mo et al. in 11 patients (16 knees) with congenital patellar dislocation in pediatric patients, and they reported promising results with this technique. Down syndrome was present in only two cases [30].

Interestingly, the more recent anatomical concepts of medial patellofemoral ligament (MPFL) reconstruction in patients with DS were not described until they were first described in 2018 by Bitar et al. [31]. They used the patellar tendon as a modification of Camanho’s MPFL reconstruction technique [9] in three children (six knees) with improved functional and radiological outcomes. The procedure also included lateral release and a medial capsulectomy. However, they added medial patello-tibial ligament reconstruction in one knee.

The current study included only immature patients with DS who were surgically treated for PF instability. In all cases, the surgical procedure included MPFL reconstruction (using a partial-thickness QT autograft), lateral soft tissue release, and the Roux-Goldthwait procedure. It had the advantage of a uniform kind of procedure that was performed by one senior surgeon on a larger number of patients. Some concomitant procedures were performed in some cases, such as loose body removal in one case, meniscal repair in two cases, V-Y quadriceps lengthening in one case, and patellar chondroplasty in one case.

Herbort et al. [32] in their biomechanical study showed that the superficial strip of the QT is broad, thin, and sheet-like; so, it closely resembles the native MPFL in morphology and biomechanics. In this study, we used a partial-thickness QT graft for MPFL reconstruction with satisfactory results. Taking only the superficial strip of the QT helps to preserve quadriceps muscle function and improve post-operative recovery and rehabilitation [16, 32]. In the current study, 10 patients out of 11 regained full quadriceps muscle power and size, while one patient had quadriceps muscle wasting (10 mm in comparison to the sound side). This patient was not completely compliant with the post-operative rehabilitation.

The anatomical study of Laprade et al. [20] reported that the average length of the MPFL was 65.2 mm. In the current study, the MPFL graft length (± SD) was 84.6 ± 5 mm (range (78–92 mm)., which was sufficient for reconstruction. Intra-operatively, we did not face any cases with short MPFL grafts. The QT is broad and can be lengthened intra-operatively, even to the double of its length, by splitting it longitudinally and reflecting one half for 180^0^. Consequently, there is no concern about the graft length when using the QT for reconstruction. There is no need for any pre-operative or intra-operative graft length measurements, provided that, when harvesting the QT graft, it should be released as proximal as possible to gain the maximum possible length of the graft.

In the current technique, we used the superficial strip of the QT, leaving its distal attachment to the patella intact. Consequently, there is no need to perform any drilling or put fixation implants in the proportionally smaller and hypoplastic patellae in children with DS, which is a surgical challenge in these patellae. We could avoid the common devastating complication of patellar fracture or violation of the articular cartilage encountered with patellar drilling in many studies [15, 33]. Parikh et al. [15] have reported in their study six (3.4%) patellar fractures in which patellar fixation was achieved through patellar tunnels.

In skeletally immature patients, the variable location and close proximity of the anatomic femoral origin of the MPFL to the femoral growth plate make it challenging to localize the MPFL femoral origin during reconstruction, with a high risk of injuring the growing growth plate if a femoral tunnel is made [34]. Seitlinger et al. [35] reported a case of femoral physis injury during MPFL reconstruction in a skeletally immature patient. Our described technique is a drill- and hardware-free technique, as we depend on soft tissue fixation at the femoral side. Consequently, we did not have any physeal injuries in this study. Comparison between our pre-operative and final post-operative radiographic measurements of the MAD showed a non-significant change; besides, all patients regained full ROM. This denotes the absence of any deformities resulting from a physeal injury.

The MPFL femoral origin has been described in most of the anatomic studies as being in the “saddle” between the medial epicondyle and the AT, or within 1 cm distal to the AT [20]. We used the AMT insertion in the adductor tubercle as an intra-operative landmark to accurately locate the femoral attachment of the native MPFL without the need for fluoroscopy. We found it a reproducible method that helps to overcome the problem of person-to-person variability in the femoral attachment point of the MPFL in skeletally immature patients. It also avoids the errors of localization when relying on radiography alone. Ziegler et al. [36], in their cadaveric study, emphasized the accuracy and importance of anatomy rather than radiography for the precise localization of the anatomic femoral origin of the MPFL.

The femoral fixation method of the MPFL in skeletal immature patients, whether static [16] or dynamic [37], is a matter of controversy, as each method has its own advantages and disadvantages. In the present study, the QT graft was passed deep to the vastus medialis obliquus muscle (VMO) and then turned around the AMT. The end of the graft was then finally fixed with a non-absorbable stitch to the thick periosteum and soft tissue just distal and anterior to the AMT insertion. In this way, we think that we had a static soft tissue femoral fixation, which would be more forgiving than the static osseous fixation and, at the same time, more anatomic than the dynamic sling fixation.

In a systematic review, Shah et al. [33] found more complications in reconstruction using bone tunnels, but suture fixation in their study had a higher rate of recurrent instability. Excellent clinical results with suture fixation were reported by Sillanpää et al. [38]. Lind et al. [39]. reported that there is no difference in outcomes between femoral soft tissue and screw graft fixation for MPFL reconstruction. They also concluded that soft-tissue femoral graft fixation does not result in inferior clinical outcomes compared with screw fixation, and it can be used safely for MPFL reconstruction. In this study, suture fixation was sufficient to fix the grafts on the patellar and femoral sides with no recurrence of dislocation.

### Limitations

There are some limitations in the current study. The study was retrospective; and non-comparative, it was not possible to evaluate patellofemoral osteoarthritis due to the short duration of follow-up. Further studies with a larger sample size and a longer duration of follow-up are needed.

## Conclusion

Our proposed technique of combined soft tissue procedures, including lateral soft tissue release, MPFL reconstruction (using a partial-thickness quadriceps tendon autograft), the Roux-Goldthwait procedure, and V-Y quadricepsplasty, was an effective method for treating patellar instability in children with DS while avoiding physeal injury and patellar fracture. Functional scores and radiological outcomes were improved.

## Data Availability

No datasets were generated or analysed during the current study.
